# Career preferences of final year medical students at a medical school in Kenya–A cross sectional study

**DOI:** 10.1186/s12909-016-0528-1

**Published:** 2016-01-11

**Authors:** Hussein Dossajee, Nchafatso Obonyo, Syed Masud Ahmed

**Affiliations:** James P. Grant School of Public Health, 68, Shahid Tajuddin Ahmed Sharani, icddr,b Building (Level-6), Mohakhali, Dhaka 1212 Bangladesh; Kenya Medical Research Institute (KEMRI)–Kilifi, P. O. Box 230–80108, Kilifi, Kenya

**Keywords:** Final year medical students, Specialization, Rural, Urban, Practice

## Abstract

**Background:**

The World Health Organization (WHO) recommended physician to population ratio is 23:10,000. Kenya has a physician to population ratio of 1.8:10,000 and is among 57 countries listed as having a serious shortage of health workers. Approximately 52 % of physicians work in urban areas, 6 % in rural and 42 % in peri-urban locations. This study explored factors influencing the choice of career specialization and location for practice among final year medical students by gender.

**Methods:**

A descriptive cross-sectional study was carried out on final year students in 2013 at the University of Nairobi’s, School of Medicine in Kenya. Sample size was calculated at 156 students for simple random sampling. Data collected using a pre-tested self-administered questionnaire included socio-demographic characteristics of the population, first and second choices for specialization. Outcome variables collected were factors affecting choice of specialty and location for practice. Bivariate analysis by gender was carried out between the listed factors and outcome variables with calculation of odds ratios and chi-square statistics at an alpha level of significance of 0.05. Factors included in a binomial logistic regression model were analysed to score the independent categorical variables affecting choice of specialty and location of practice.

**Results:**

Internal medicine, Surgery, Obstetrics/Gynaecology and Paediatrics accounted for 58.7 % of all choices of specialization. Female students were less likely to select Obs/Gyn (OR 0.41, 95 % CI =0.17-0.99) and Surgery (OR 0.33, 95 % CI = 0.13-0.86) but eight times more likely to select Paediatrics (OR 8.67, 95 % CI = 1.91-39.30). Surgery was primarily selected because of the ‘perceived prestige of the specialty’ (OR 4.3 95 % CI = 1.35-14.1). Paediatrics was selected due to ‘Ease of raising a family’ (OR 4.08 95 % CI = 1.08-15.4). Rural origin increased the odds of practicing in a rural area (OR 2.5, 95 % CI = 1.04-6.04). Training abroad was more likely to result in preference for working abroad (OR 9.27 95 % CI = 2.1-41.9).

**Conclusions:**

The 4 core specialties predominate as career preferences. Females are more likely to select career choices due to ‘ease of raising a family’. Rural origin of students was found to be the most important factor for retention of rural health workforce. This data can be used to design prospective cohort studies in an effort to understand the dynamic influence that governments, educational institutions, work environments, family and friends exert on medical students’ careers.

**Electronic supplementary material:**

The online version of this article (doi:10.1186/s12909-016-0528-1) contains supplementary material, which is available to authorized users.

## Background

Kenya is a sub-Saharan country situated in East Africa with an estimated Gross National Income per capita in purchasing power parity of US$ 1160 (GNI per capita in PPP dollars) [1]. Out of a total population of 43 million; 36 million (82 %) people live in the rural areas [1]. The Health Workforce is defined by the World Health Organization (WHO) as “*all people engaged in actions whose primary intent is to enhance health*” [2]. Kenya is one of the 57 countries with a Human Resource for Health (HRH) crises [2]. It has a physician to population ratio of 1.8/10,000 population [1]. WHO recommends a ratio of 23/10,000 for adequate health care services [3]. In Kenya it is estimated that 52 % of physicians work in urban areas, 6 % in rural areas and 42 % in peri-urban areas [4]. In 2010, there was a 41 % shortage of General Medical Doctors [4]. In the year 2014, The Ministry of Health published a report showing a deficit in multiple specialties. There were a total of five hundred and one physicians in the country with 33 % in the major towns of Nairobi, Mombasa and Kisumu. Forty four percent (44 %) of Pediatricians are within 5 major urban centers. Out of a total of twenty one (21) Dermatologists in the country, 43 % are in Nairobi. Other fields of specialization have a similar skewed distribution [5]. With this shortage and mal-distribution of the health workforce, Kenya will not achieve any of the eight Millennium Development Goals (MDG’s) [6]. The number of health professionals in an area and more importantly their distribution has been shown to have a direct impact on health outcomes [7]. This mal-distribution, work force shortage and imbalance in skill-mix exist within poor and rich countries [8, 9]. Multiple factors affect the demand and supply of health care workers in a country. Factors influencing the demand include demographic characteristics of population, consumer preferences, financing and globalization. This is particularly true for medical doctors [10]. Supply of health workers can be affected by choice of professional training. Individual choice of specialization/training may not meet the market demands resulting in shortage of labour in some sectors and surplus in others [10]. Other supply side factors include migrations and regulations. Health workers migrate in search of better work conditions and remuneration, social recognition and opportunities for training [11, 12]. International migration has resulted in depletion of the medical workforce in sub-Saharan Africa. In the report published for six African countries in 2003, doctors who expressed willingness to emigrate ranged from 26 % in Uganda to 68 % in Zimbabwe [13]. The most common reason cited for emigration was better remuneration. Approximately 51 % of Kenya’s total population of physicians has emigrated between the years 1998–2003, citing better remuneration and opportunities for career advancement [14]. Apart from affecting health care delivery, economic loss incurred by a country is estimated at US$ 1.8 million per doctor who emigrates [15]. An additional factor mentioned in this report which affects both demand and supply was the presence of a stable socio-political environment [13]. In the study carried out by Zurn et al., [10] lack of drugs and amenities have also been cited as a reason why specialist trained doctors prefer urban practice, especially surgeons. Regulations which are set by government or professional bodies can restrict supply of health workers by limiting entry into the profession. This applies primarily to nurses, doctors and dentists [16]. In an attempt to understand how to retain rural health workforce of doctors a study in Ghana was carried out [17]. This study compared rural versus urban origin of students and whether this affected future location of practice. The results of this study suggest that students with rural origin were more likely to return to practice in rural areas. In this study international working experience or training was more likely to result in avoidance of rural practice. This may have policy implications on selection of students for medical schools especially where rural–urban maldistribution of doctors exists [17].

Another important factor identified from a study carried out in South Africa was the role of religious beliefs which seemed to influence the selection of rural location [18]. This qualitative study did not attempt to explore the theme further but the religious aspect was reported as being recurrently identified from multiple in-depth interviews

In Kenya, Family Medicine as a specialty choice has often been neglected. Doctors specialized in Family Medicine are trained to manage common ailments for all family members and for emergency procedures in order to stabilize a patient’s condition before transferring to the nearest health facility. Evidence suggests that Family Medicine when combined with rural origin of students may result in greater retention of doctors in rural areas with improved services [19].

The role of rural clinical service during undergraduate medical training has been studied in order to provide solutions to retention of rural doctors. It was found to play a positive role in attracting medical doctors to rural practice [20].

Medical school tuition fees are expensive in most countries, compelling students and their families to take loans. Debt incurred as a result of these expenses and whether it influences future career options and location of practice has been questioned. A study from the United States (US) suggests that this may be true [21]. The perceived income from different specialties and perceived prestige of a field of specialization may also influence career options [22].

In an attempt to understand the choice of specialization and career pathways of medical students, a study was carried out at the University of Nairobi (UoN), Kenya. This study suggests that gender-based differences exist in influencing specialty preferences among Kenyan medical students in pre-clinical and clinical years. Males preferred surgical fields whilst females had preference for Pediatrics, Pathology, Obstetrics/Gynaecology and Radiology. This was attributed to prestige, presence of role models for males and a controllable lifestyle due to family responsibility for females [23]. Similar findings were noted in studies carried out in Malawi [24] and South Africa [25].

There is lack of data on career preferences of medical students in Kenya. Only one study has looked at factors affecting career preferences of medical students at UoN [23]. However, this study compared clinical and pre-clinical students. It did not confound for the fact that pre-clinical students have no exposure to clinical work and 3rd, 4th and 5th year students have different levels of exposure. It is more appropriate to study the final year students who are on verge of entering the profession and have started giving thought to this issue. In this study, only final year students were studied. The role of self-sponsorship versus government subsidy as a determinant of career choices for medical students has not been studied in Kenya. The previous study did not determine association of rural versus urban origin and choice of location for practice, which this study attempted to do. The objectives of this study were to compare socio-demographic characteristics of final year medical students by gender; explore specialization preferences of final year medical students by gender; explore factors affecting the choice of selected specialty and choice of location for practice.

## Methods

This was a descriptive cross-sectional study. It was carried out at the University of Nairobi’s School of Medicine, an urban setting, in Nairobi County, Kenya. This School is affiliated to Kenyatta National Hospital, Kenya’s largest tertiary health facility. Students were selected from the final year of medical school in 2013. Simple random sampling, using the class register as sampling frame, was carried out at the medical school. The size of this sampling frame was 260 students. Alternate students on the register were selected. This register is arranged according to registration number. Sample size was calculated for a finite population-without replacement after applying a population correction factor [26]. With a 95 % confidence level and 5 % margin of error, sample size was calculated at 156 students. A 10 % allowance for non-response estimated sample size at 172 students. Data was collected using a close ended self-administered questionnaire in English (see Additional file 1). Ten percent of sample size (*n* = 18) was selected for pre-testing of questionnaire. Data was analyzed to correct ambiguities in questionnaire before distribution to sampled population. For religious affiliation the option of ‘others-Jewish, Sikh, Traditional African’ was deleted as it was not selected in pre-testing. Psychiatry was added to career choices. Minor adjustments were made to language construct. This questionnaire was adapted from four published studies which used similar questions modified to local context [23, 24, 27, 28]. Questions with a Cronbach’s correlation coefficient (α) greater than 0.7 were utilized for this study. Students who were undertaking Bachelor of Medicine and Bachelor of Surgery (MBBS/MBChB) as the first degree course, willing to participate and resident/citizen of Kenya were included. Students who were absent or who had repeated or failed any year of medical school were excluded from this study. The outcome variables were ‘Choice of specialization’-categorized as discrete variables into fifteen (15) specialties and two open options (i.e. ‘Not decided’ and ‘Non-medical’ (intention to leave clinical medicine) and ‘Location of practice’-categorized into rural, urban or international migration. The Independent variables included socio-demographic data (age, gender, marital status, rural–urban origin, religious affiliation), student sponsorship (self-sponsored or government subsidy) and Parents/Guardians profession. Factors affecting ‘choice of specialty’ which had a list of eighteen (18) independent factors and factors affecting choice of ‘location to practice’ which had fourteen (14) factors included as independent variables. These were answered as either ‘Yes’, ‘Maybe’ or ‘No’ (see Additional file 1). Data was entered into Excel^©^ spreadsheets and transferred to statistical package STATA 12^©^ for analysis. This data was entered in duplicate and compared for concordance. Discordant entries were resolved by tracing original questionnaire from the assigned serial number. The Chi-square (χ^2^) test was used to determine the presence of statistically significant differences by gender for socio-demographic characteristics. Choice of specialization was analyzed and compared across gender category by means of Chi square. Independent variables that were identified to play a key role in literature review were selected from factors affecting specialty choice and factors affecting location of practice and analyzed by gender before inclusion into multivariate analysis. A binomial regression model was developed to obtain the coefficients with 95 % confidence interval (95 % CI). Specialization choices were grouped into ‘controllable’ and ‘non-controllable’ lifestyles. ‘*Controllable lifestyle specialties*’ were defined as those that allow the physician to control the number of hours devoted to practicing the specialty. ‘*Non*-*controllable lifestyle specialties*’ were defined as those that do not allow a physician to control number of hours devoted to specialty [29, 30]. Controllable lifestyle included the following specialties–Academic Medicine, Basic Sciences, Family medicine/ General practice, Pathology, Public health, Radiology and Psychiatry.

Non-controllable lifestyle included the following specialties–Anaesthesia, Ears Nose Throat (ENT), Internal medicine, Obstetrics/Gynaecology, Ophthalmology, Orthopaedics, Paediatrics and Surgery. Options of ‘Not-decided’ and ‘Non-medical’ were analyzed separately and were not included as part of this variable. The independent variable groups for factors affecting choice of specialization (18 in total) and factors affecting choice of location (14 in total) were analyzed by means of factor analysis. Factors that scored an eigen value greater than 1.0 were retained. Regression models were created for addition of other independent variables such as age, gender, marital status and payment of tuition.

### Ethical consideration

Before commencement of data collection, ethical approval was taken from Ethical review committee of the BRAC Institute of Global Health. Authorization was sought and obtained from the administrative authorities at the University of Nairobi -School of Medicine.^1^ Voluntary verbal and written consent was obtained from each study participant.

## Results

### Socio-demographic information

All questions from the piloted questionnaires with a Cronbach’s alpha coefficient of ≥ 0.7 were included in the questionnaire. A total of one hundred and fifty five (155) questionnaires were returned which calculated to a response rate of 87.1 %. One hundred and fifty one (151) questionnaires were completely filled for every item resulting in 97.0 % complete data. Except where stated, data from all 155 questionnaires was included in the data analysis.

Male to female ratio of students was approximately 1:1. The mean age for male students (*n* = 76) was 24.84 ± 1.08 years with a range of 23-29 years. The mean age for female students (*n* = 79) was 24.31 ± 1.35 years with a range of 22–31 years.

Female students were four times more likely to have originated from an urban area than male students. Female students were less likely to be government sponsored when compared to male students (Table 1).

**Table 1:** Bivariate analysis of background characteristics of students by gender

Variable	Gender					
	M (*n* = 76)		F (*n* = 79)		Odds ratio	*p*-value
	n	%	n	%	(95 % CI)	
Marital status						
Single	72	94.7	70	88.6	1.00	
Married	4	5.3	9	11.9	1.44 (0.68-7.86)	0.179
Location majority of life lived						
Rural	20	26.3	6	7.6	1.00	
Urban	56	73.7	73	92.4	4.34 (1.68-4.67)	^a^0.003
Religious affiliation						
Christian	56	73.7	67	84.8	1.0 (0.84-1.71)	0.144
Muslim	15	19.7	9	11.4	0.5 (0.20-1.23)	0.133
Hindu	4	5.3	2	2.5	0.5 (0.092-2.73)	0.144
None	1	0.01	1	0.01	1.0 (0.06-15.98)	0.144
Profession Mother/female guardian						
non-medical	68	89.5	70	88.6	1.00	
Medical	8	10.5	9	11.4	1.09 (0.40-2.99)	0.863
Profession Father/male guardian						
non-medical	68	89.5	71	89.9	1.00	
Medical	8	10.5	8	10.1	0.96 (0.34-2.69)	0.935
Tuition fees						
self-sponsored	27	35.5	50	63.3	1.00	
government subsidy	49	64.5	29	36.7	0.32 (0.16-0.61)	^a^0.001

^a^significant at 0.05 level of alpha. *n* number of students in that category with % allocated proportion

Odds Ratio expressed as female: male

### Specialization choices

Male students in general had an orientation to surgical specialties and female students towards the non-surgical fields. Female students were less likely to select Obs/Gyn, Surgery or Orthopaedics. Females were 8 times more likely to select Paediatrics than male students. Non- clinical specialties such as Academic medicine, Basic science were poorly selected by both genders (Table 2).

**Table 2:** First preference for specialization as distributed by gender

1^st^ Specialty	Male		Female		OR (95 % CI)	
	n	%	n	%		*p*-value
Anaesthesia	0	0	4	5.06	N/A	
Academic medicine	2	2.63	2	2.53	0.96 (0.13-7.00)	0.969
Basic science	1	1.32	0	0	N/A	
ENT	1	1.32	6	7.59	6.16 (0.72-52.4)	0.096
Family medicine/GP	1	1.32	2	2.53	1.94 (0.17-21.94	0.589
Internal Medicine	10	13.16	13	16.46	1.3 (0.53-3.17)	0.564
Obs/Gyn	18	23.68	9	11.39	0.41 (0.17-0.99)	^a^0.044
Ophthalmology	2	2.63	4	5.06	1.97 (0.35-11.10)	0.441
Orthopaedics	14	18.42	1	1.27	0.05 (0.01-0.44)	^a^0.006
Paediatrics	2	2.63	15	18.99	8.67 (1.91-39.30)	^a^0.005
Pathology	0	0	1	1.27	N/A	
Public health	2	2.63	3	3.8	1.46 (0.23-8.99)	0.681
Radiology	2	2.63	5	6.33	2.5 (0.47-13.29)	0.382
Surgery	17	22.37	7	8.86	0.33 (0.13-0.86)	^a^0.020
Psychiatry	0	0	0	0	N/A	
Not decided	4	5.26	6	7.59	1.48 (0.47-5.46)	0.555
Non-medical (leave medicine)	0	0	1	1.27	N/A	
Total	76	100	79	100		

^a^significant at alpha level 0.05, *N*/*A* not applicable, *n* number of students who selected a particular specialty with % proportion

Odds Ratio indicated as female: male

Internal Medicine, Surgery, Paediatrics and Obs/Gyn in total, accounted for 58.7 % (*n* = 91) of all specialty choices (Table 2).

As a second choice of specialization, Pediatrics remains a preferred choice for female students. Male students still predominate in Surgery, Internal medicine and Orthopaedic surgery (Table 3).

**Table 3:** Bivariate analysis for second choice of specialization as distributed by gender

2^nd^ Specialty	Males		Females		OR (95 % CI)	
	n	%	n	%		*p*-value
Anaesthesia	3	3.95	6	7.59	2.00 (0.48-8.30)	0.34
Academic medicine	1	1.32	2	2.53	1.95 (0.17-21.93)	0.589
Basic science	0	0	1	1.27	N/A	
ENT	4	5.26	5	6.33	1.21 (0.31-4.71)	0.772
Family medicine/GP	3	3.95	4	5.06	1.30 (0.28-6.00)	0.739
Internal Medicine	5	6.58	3	3.8	0.56 (0.13-2.43)	0.439
Obs/Gyn	6	7.89	4	5.06	0.62 (0.16-2.30)	0.477
Ophthalmology	0	0	3	3.8	N/A	0.086
Orthopaedics	9	11.84	0	0	N/A	
Paediatrics	5	6.58	16	20.25	3.60 (1.24-10.41)	^a^0.018
Pathology	1	1.32	0	0	N/A	
Public health	8	10.53	12	5.19	1.52 (0.59-3.96)	0.389
Radiology	3	3.95	4	5.06	1.30 (0.28-6.00)	0.738
Surgery	19	25	10	12.66	0.43 (0.18-1.01)	0.053
Psychiatry	1	1.32	0	0	N/A	
Not decided	5	6.58	6	7.59	1.17 (0.34-3.99)	0.805
Non-medical (leave medicine)	3	3.95	3	3.8	0.96 (0.18-4.92)	0.961
Total	76	100	79	100		

^a^Significant at alpha level 0.05, *N*/*A* not applicable, *n* number of students selecting a particular specialty with % proportion

Odds ratio represents female: male preference for a particular specialty

There was an increase in proportion of students from 1.27 % (*n* = 1) to 7.75 % (*n* = 6) for those who intend to leave medicine (‘non-medical’). This represents a fivefold increase in number of students who might wish to leave a medical career if their first choice is not successful (Tables 2 & 3).

The top 4 specialties selected as a first choice (Table 2) account for 43.9 % of all choices selected from the second choice of specialty with Public health accounting for another 17.5 % (Table 3).

Both Tables 2 & 3 show a preference for clinical specialties versus non-clinical specialties. Academic medicine, Basic sciences and Psychiatry are poorly selected as first and second choice of specialization.

Re-grouping of specialty choices into controllable and non-controllable lifestyle did not show any statistically significant difference in selection by gender. (Table 4)

**Table 4:** Bivariate analysis for re-grouping of specialization choices according to lifestyle, with comparison by gender

Grouping of specialty choice by lifestyle	Males		Females		OR (95 % CI)	
	n	%	n	%		*p*-value
1st choice of specialty						
Controllable lifestyle	8	11.1	13	18.1	0.67 (0.18-2.46)	0.543
Non-controllable lifestyle	64	88.9	59	81.9		
2nd choice of specialty						
Controllable lifestyle	17	25.0	23	32.9	0.85 (0.25-2.94)	0.807
Non-controllable lifestyle	51	75.0	47	67.1		

Significance level of alpha 0.05, *n* number of students who chose specialty with their proportion, Options of ‘not-decided’ and ‘non-medical’ were excluded from this analysis

Odds ratio compares female: male selection of non-controllable versus controllable lifestyle

### Factors affecting choice of specialization

Eighteen (18) factors were analyzed by bivariate analysis for the 4 major specialties selected of Internal medicine, Surgery, Obs/Gyn and Paediatrics. For the analysis below all students who had ‘Not-decided’ on a career choice or who intended to leave medicine (‘non-medical’) were excluded.

The factors that were significant for selecting Surgery as a career over the other 3 specialty choices were the ‘perceived prestige of the specialty’ (OR 4.3 95 % CI = 1.35-14.1, *p*-value 0.014) and ‘ease of raising a family’ (OR 0.32; 95 % CI = 0.11-0.90, *p*-value 0.030).

Those who selected Paediatrics as a career selected the following factors as being influential to their choice of career. ‘Ease of raising a family’ (OR 4.08 95 % CI = 1.08-15.4; *p*-value 0.038) and ‘Job opportunity’ (OR 11 95 % CI = 1.18-102.0; *p*-value 0.035). Internal medicine was selected because of ‘perceived income potential’ (OR 0.22 95 % CI = 0.08-0.62; *p*-value 0.004). Obs/Gyn was selected because of ‘perceived income potential’ (OR 5.16 95 % CI = 1.13-23.4; *p*-value 0.034).

Acceptable hours of practice, appraisal of own skills and aptitude, desire to provide community service, ease of entry into residency, encouragement by teaching staff, family influence, gender distribution in specialty, health promotion & prevention opportunity, illness in family member, illness in self, intellectual challenge, length of residency, peer pressure and role model were not found to be significant factors from the list of 18 variables.

Binomial logistic regression with all factors was carried out after factor analysis for the 18 independent categorical variables. Outcome variable was the choice of specialization grouped according to lifestyle. None of the factors were found to be significant (Table 5).

**Table 5:** Binomial logistic regression for factors affecting choice of specialization when specialties categorized into non-controllable versus controllable lifestyles

Lifestyle (non-controllable versus controllable)	Odds ratio	(95 % CI)	*p*-value
Sex (female vs male)	1.89	(0.22-15.86)	0.558
Marital status (married vs single)	1	-	-
Spent most of life (urban vs rural)	1.57	(0.10-24.59)	0.747
Religion (base category Christianity)			
Muslim	2.89	(0.15-56.10)	0.483
Hindu	1	-	-
None	1	-	-
Profession of mother/female guardians (non-medical vs medical)	1	-	-
Profession of father/male guardian (non-medical vs medical)	1	-	-
Fees payment (government vs self)	0.28	(0.03-2.77)	0.275
Factors affecting choice of specialization (base outcome is ‘No’)			
Maybe	0.32	(0.02-6.24)	0.452
Yes	0.47	(0.03-7.56)	0.594

Significance level alpha 0.05 the variable labelled ‘factors affecting choice of specialization’ was generated after factor analysis for the 18 independent categorical variables on questionnaire [see Additional file 1- section B2]

Odds ratios compare non-controllable to controllable lifestyle

### Factors affecting location of practice

Bivariate analysis was carried out for factors affecting location of practice. Rural origin increased the odds of practicing in a rural area (OR 2.5, 95 % CI = 1.04-6.04). Training abroad was more likely to result in preference for working abroad (OR 9.27 95 % CI = 2.1-41.9). Access to social and family networks was found to be more important for female students versus male students. Quality of workplace, Remuneration, Political stability and safety/security were analyzed separately as proportions due to importance attached to them in literature review when location of practice is considered. Quality of workplace stood out as a key factor with approximately 73 % of respondents selecting it. Factor analysis was carried out for the fourteen (14) independent variables affecting ‘location of practice’.

Binomial logistic regression was carried out with this variable and all other variables affecting location of practice. None of the independent variables was found to be statistically significant (Table 6).

**Table 6:** Binomial logistic regression analysis for factors affecting ‘Location of practice’

Location of practice (urban versus rural)	Odds Ratio	(95 % CI)	*p*-value
Majority of life spent (urban vs rural)	1.89	(0.75-4.81)	0.177
Intention to train (Abroad vs Kenya)	1.74	(0.11-28.37)	0.697
Gender (female vs male)	1.63	(0.74-3.63)	0.226
Marital status (married vs single)	2.80	(0.33-23.91)	0.346
Religious affiliation (base category Christianity)			
Muslim	2.07	(0.63-6.87)	0.230
Hindu	1	-	-
None	1	-	-
Factors affecting location of practice (base outcome ‘No’)			
Maybe	1.73	(0.68-4.39)	0.253
Yes	1.36	(0.55-3.36)	0.501

The variable labelled ‘factors affecting location of practice’ was generated after factor analysis of 14 independent variables on questionnaire. [see Additional file 1- section C4]

Odds ratios compare urban versus rural practice

## Discussion

Internal medicine, Surgery, Obs/Gyn, Orthopaedics and Paediatrics were the major choices selected by the students. These findings were similar to choices made by students in other low and middle income countries [28, 31]. Whether the education system plays a role in this selection needs to be determined. Often, medical school cannot expose a student to the vast variety of medical career options and training is limited to the traditional core specialties. A study carried out in US suggest that a medical career advisory service may help students choose their careers based on their skills, interest as well as aid with exploratory postings to different fields of specialization during elective term. This might help to broaden career options and match students with suitable careers [32]. Quality of workplace and remuneration were mentioned by students as important factors for career choice, but the difference was not significant. Both have been found to be important factors in other studies [9, 14, 33].

Academic Medicine and Basic science are poorly selected despite these professions providing the core of teaching in the medical education system. The current ratio of lecturer to students stands at 1:19, against a recommended ratio by the Commission for Higher Education, Kenya at 1:7 [34]. This shortage may impact on the quality of medical education provided [35]. Basic sciences have been considered as an ‘inferior’ career as compared to clinical medicine and remuneration and prestige attached to this career is often lacking [9, 23, 35]. Addressing this issue as a means of resolving the shortage of lecturers needs to be attended to in order to prevent compromising medical education standards. Urban practice may be partly explained by the fact that all medical universities in Kenya are in urban areas. Role modelling was not selected by students as an influential factor, even though it was important in other studies [27, 36, 37]. Psychiatry was not selected by any student as a primary choice but selected by 1 student as a secondary choice. The reasons behind students neglecting this specialty need to be explored further. Kenya has a poor Psychiatrist to population ratio estimated at 1: 590,000. This ratio drops to 1:4,000,000 in some areas of the country [38]. This is important because poverty and associated conditions of unemployment, low educational level, deprivation and homelessness are twice as likely to lead to mental disorders such as depression [39]. Family Medicine was poorly selected as a 1st choice of specialty. It is becoming an important specialty worldwide in an effort to reduce the rural–urban maldistribution of doctors and to cope with demographic changes in populations with increase in elderly populations particularly in rural areas. Despite being promoted as a solution to this problem, uptake has been difficult in both low and high income countries [40]. To retain rural supply of doctors, it has been found to be important to train students with a rural origin and site universities in rural areas [17, 18, 20]. When rural origin was compared with intention to practice in rural areas there was a statistically significant association in this study which concurs with the studies cited above. To increase interest for practice in rural areas, work done in Australia suggests that placement of students in rural hospitals as part of medical training may increase retention and desire for practice in rural areas. This was regardless of whether a student was of rural or urban origin [20].

Studies carried out in different countries of low and high income suggest a gender bias in choice of specialization. Females have a tendency to select specialties such as Obs/Gyn, Paediatrics Anaesthesia and Radiology. Males have a tendency to select Surgery, Internal Medicine and Orthopaedics [30, 33, 41].

Similar findings were obtained in this study with exception of Obs/Gyn where more male students selected the specialty. The reasons behind more males preferring this specialty may need further exploration but in this study ‘job opportunity’ and ‘perceived income potential’ were cited as major reasons by male respondents. Despite Surgery being selected more by males, some females did select it as a career choice with ENT & Ophthalmology (both involving surgical skills) being selected more by females. This might imply that Surgery as a male dominated field might actually be on the decline [42]. After factor analysis was carried out, it was found that none of the independent variables were significant for selecting field of specialization. It is possible that the sample size is too small and a larger study incorporating other universities may show different results.

When specialization was grouped into controllable and non-controllable lifestyles and compared across gender, there was no significant difference between males and females. This classification has been challenged as being too generalized, but studies have shown that it is a good way to compare the multiple medical specialties that exist which may not strictly fall under ‘clinical’ or ‘non-clinical’ [29, 30, 43].

Despite not being statistically significant, it is of concern that the number of students who selected the option of leaving medicine if their first choice was not successful increased by almost 5 times. Annually, Kenya loses about 8 % of its doctors in the public sector through resignation, retirement and death. Any reduction in the number of graduates becomes very important when the capacity to train them is limited [44]. Studies might need to be carried out to determine the reasons behind why students would train for 5 years in medical school and then leave the profession. A study carried out in New Zealand to determine some of these causes found that being humiliated or degraded by teaching staff was the most commonly mentioned reason, followed by racial discrimination and gender harassment [45]. There are no studies in Africa supporting this. Whether similar reasons prevail in the Kenyan university needs to be determined.

In this study religious affiliation was not found to have significant association with choice of specialty or choice of location as suggested by Couper et al. [18].

International migrations have resulted in depletion and shortages in the workforce of low income countries [13]. In this study 88 % (*n* = 136) of students intend to practice in Kenya after specialty training. However, 75 % (*n* = 103) students who intend to practice in Kenya have a preference for Urban practice. Incentives to encourage rural practice need to be created to retain the rural workforce and reduce rural–urban maldistribution.

Based on the binomial logistic regression for factors affecting choice of specialization, mother/female guardian’s profession was not found to be statistically significant. Multiple studies suggest that mothers play a role in selection of career [46, 47]. Fathers/male guardian’s profession was not found to be a significant factor in this study. Neither was family influence and peer pressure. It has been suggested that parents role in influencing a child’s career may take place in high school. Mothers play a greater role but fathers may influence their son’s choice of career. Peer influence especially in high school and societal perception of a career are suggested as other factors [48, 49]. When binomial logistic regression was carried out for factors affecting choice of location for practice, none of these factors were found to be significant. It is possible that these factors may be classified too broadly. Sub-analysis by re-grouping of factors may yield information that was overlooked by this broader classification.

The limitation of this study is that it is a cross sectional study. Career preferences may change over time. A prospective cohort study following these students through internship and assessing their career preferences at the end might yield valuable information on trends in career choices influenced by internship.

Final year students were studied at one public university located in Nairobi. It is not possible to generalize findings to the other three public universities and one private medical university. A larger study involving all universities would be required to draw conclusions as each of these universities are located in different parts of the country, taught by different faculty and have autonomy over different aspects of the syllabus.

## Conclusions

Gender differences exist in selection of specialty with male preference towards surgical specialties. ‘Perceived income potential’ was the most important reason for selecting a particular specialty. Urban practice is preferred to rural practice. This study helps to understand the fact that choice of medical specialties and location of practice is multi-dimensional and multi-factorial and if maldistribution of doctors requires a solution it must involve medical students, their parents/guardians, universities, the communities involved and the government.

Kenya is in a transition phase where the central government is devolving healthcare to county governments. Education, remuneration and terms of service will be negotiated at county level. Prospectively following this cohort of students would provide valuable information as to how governance can affect careers of doctors.

## Additional file

Additional file 1: Questionnaire used in the study (DOCX 21 kb)

## Abbreviations

ENT: ears nose throat surgery
GNI per capita PPP dollars: gross national income per capita in purchasing power parity of US$
HRH: human resource for health
MBChB/MBBS: bachelor of medicine and bachelor of surgery
MDG’s: millennium development goals
Obs/Gyn: obstetrics/gynaecology
UoN: University of Nairobi
US: United States
WHO: world health organization

## Footnotes

1 National Council for Science and Technology. Procedures and Guidelines for Research Authorization in Kenya. Nairobi: Ministry of Higher Education, Science and Technology; 2009: 5–6. http://finance.uonbi.ac.ke/sites/default/files/centraladmin/finance/ResearchClearanceGuideline.pdf. Accessed 20^th^ October, 2013.
