# Supplementary material for: Career preferences of final year medical students at a medical school in Kenya–A cross sectional study
Source: BMC Med Educ. 2016 Jan 11;16:5. doi: 10.1186/s12909-016-0528-1 (PMC4709906; doi:10.1186/s12909-016-0528-1)
Supplement: Additional file 1: — Questionnaire used in the study (DOCX 21 kb) [file 12909_2016_528_MOESM1_ESM.docx]

**Additional file 1: Questionnaire used in the study**

Questionnaire on ‘Factors affecting career preferences of final year Medical students in Kenya’

Serial No: *(to be filled by researcher) __________*

**Section A: Background Information** Date: *(dd/mm/yy)* ___________

1. Age in years_____________
2. Gender: ☐Male ☐ Female
3. Marital status: ☐ Single ☐ Married
4. Where have you spent most of your life: ☐ Rural ☐ Urban/Small town
5. Religious affiliation: ☐ Christian ☐ Muslim ☐ Hindu
6. Profession of Mother/ Female guardian: ☐ Medical ☐ Non-medical
7. Profession of Father/Male guardian: ☐ Medical ☐ Non-medical
8. Payment of tuition fee; ☐ Self-sponsored ☐ Government subsidy/sponsored

**Section B: Choice of Specialization**

1. What are your top two (2) choices for specialization? Please rank your preferred choices as 1 and 2 from the list given below

| **Specialty choice** | **Mark choice 1 & 2 only** |
| --- | --- |
| Anaesthesia |  |
| Academic Medicine (Research and Teaching) |  |
| Basic Science (Anatomy, Biochemistry, Physiology) |  |
| ENT (Ear Nose Throat) |  |
| Family Medicine/ General Practice |  |
| Internal medicine (includes sub-specialties) |  |
| Obstetrics and Gynaecology |  |
| Ophthalmology |  |
| Orthopaedics |  |
| Paediatrics (includes sub-specialties) |  |
| Pathology (includes sub specialty e.g Clinical Chemistry, Immunology, Microbiology) |  |
| Public health |  |
| Radiology |  |
| Surgery (includes all sub-specialties) |  |
| Psychiatry |  |
| Not decided **(if selected skip section B2, go to C)** |  |
| Non- medical (leave clinical or laboratory based medicine) **(if selected, End the questionnaire here)** |  |

1. Did any of the following factors influence your choice of specialization? Please tick ‘Yes’ ‘Maybe’ or ‘No’(select one choice for each statement)

| **Factors affecting choice of specialty** | **Yes** | **Maybe** | **No** |
| --- | --- | --- | --- |
| Acceptable hours of practice |  |  |  |
| Appraisal of own skills and aptitude |  |  |  |
| Desire to provide community service |  |  |  |
| Ease of entry into residency training program |  |  |  |
| Ease of raising a family |  |  |  |
| Encouragement by teaching/clinical staff |  |  |  |
| Family influence/pressure |  |  |  |
| Gender distribution in specialty |  |  |  |
| Health promotion and prevention opportunities |  |  |  |
| Illness in family member |  |  |  |
| Illness in self |  |  |  |
| Intellectual challenge in specialty |  |  |  |
| Job opportunities/job security |  |  |  |
| Length of residency |  |  |  |
| Peer pressure (by fellow students/friends) |  |  |  |
| Perceived Income potential |  |  |  |
| Perceived prestige of specialty |  |  |  |
| Role model/mentorship in the specialty selected |  |  |  |

**Section C: Location of Practice:**

1. Where do you intend to practice after internship? ☐ Rural ☐ Urban/Small town
2. Where do you intend to obtain further training? ☐ Kenya ☐ Abroad
3. Where do you intend to spend majority of your working life after specialist training?

☐ Kenya ☐ Abroad

1. Which of the factors listed below will influence your choice for location of practice? Please tick ‘Yes’ or ‘ Maybe’ or ‘No’ (select one choice for each statement)

| **Factor affecting choice of location** | **Yes** | **Maybe** | **No** |
| --- | --- | --- | --- |
| Access to social and family networks |  |  |  |
| Adventure & recreational opportunities |  |  |  |
| Availability of other specialists |  |  |  |
| Community belonging |  |  |  |
| Opportunities for children |  |  |  |
| Opportunities for own continuing education |  |  |  |
| Opportunities for partner/spouse |  |  |  |
| Political stability |  |  |  |
| Quality of work facilities |  |  |  |
| Registration and Accreditation procedure |  |  |  |
| Remuneration |  |  |  |
| Rural lifestyle |  |  |  |
| Safety and security issues |  |  |  |
